# Children’s Independent Mobility: Current Knowledge, Future Directions, and Public Health Implications

**DOI:** 10.3390/ijerph15112441

**Published:** 2018-11-01

**Authors:** Isabel Marzi, Anne Kerstin Reimers

**Affiliations:** Institute of Human Movement Science and Health, Faculty of Behavioral and Social Sciences, Chemnitz University of Technology, 09111 Chemnitz, Germany; anne.reimers@hsw.tu-chemnitz.de

**Keywords:** physical activity, health promotion, determinants, trends, measurements, active travel, health, environment

## Abstract

Environmental changes significantly impact health behavior. Active travel behavior is mostly affected by increasing motorization, urban sprawl, and traffic safety. Especially for children, active and independent travel can contribute to physical activity, social and motor development, and other health-related outcomes. A reduced number of children engaging in independent mobility over the last 20 years demanded researchers to further examine the construct of children’s independent mobility. By examining relevant literature, this narrative review aims to provide the current state of knowledge on children’s independent mobility, and identify future directions in research, as well as practical implications. From a public health perspective, considering children’s independent mobility in intervention programs is recommended, since it is associated with numerous health and environmental benefits. To develop interventions, multilevel socio-ecological influences on children’s independent mobility are widely examined; however, evidence is limited due to heterogeneous measurements and a lack of high-quality prospective studies. To oppose the decline in children’s independent mobility, further analysis using comparable measures is needed to understand the determinants of children’s independent mobility and to enable international comparison.

## 1. Introduction

Physical inactivity is identified as a major risk factor for global mortality [1]. However, less than 20% of children worldwide meet the recommended physical activity guidelines of the World Health Organization [2]. In a representative study in Germany, the number of children participating in organized sport activities (e.g., sport clubs) increased from 2003 to 2012, while unorganized physical activity, as well as overall physical activity, decreased significantly over time [3]. Hence, in health promotion, considering unorganized leisure-time physical activities—which includes children’s independent mobility (CIM)—is an important step to take. Various studies showed that children who walk or cycle to school independently are significantly more physically active [4] and meet the physical activity recommendation more often [5] than children who are driven to school. Thus, independent mobility is an important contributor to physical activity and health in children. Since researchers identified a significant decline in children traveling around the neighborhood on their own [6,7,8], the examination of children’s independent mobility and its determinants gained importance in the field of public health.

Altered societal and living conditions are considered as reasons for changes in children’s travel behaviors [9,10,11]. Built environment progress and an increased motorization characterize actual physical environmental changes which restrain children to be independently mobile [10,12]. In addition, increased safety concerns of parents [13,14], higher amounts of organized leisure activities [12], and family routines [15], as well as longer commuting distances caused by urban sprawl [16], limit children’s mobility opportunities and activity spaces [9,17]. Further benefits of children being independently mobile instead of motorized are low monetary costs for traveling and environmental sustainability through reduced car use, traffic volume, and air pollution.

All these aspects implicate the identification of potential modifiable attributes to establish effective (health promotion) interventions. Following the goal of making the environment more child-friendly and to promote CIM, researchers of different fields (e.g., urban planners, health scientists, and sociologists) considered the construct of CIM [18,19,20,21]. 

The aim of this narrative review is to provide a current state of research on CIM, practical implications, and future directions in research. National and international articles were reviewed considering indicators, measurements, prevalence, and trends of CIM. Additionally, studies which evaluated either the relationship between CIM and health outcomes or the determinants of CIM were searched to establish a comprehensive narrative overview on this topic.

## 2. Children’s Independent Mobility—Indicators and Measurements

**Figure 1:**
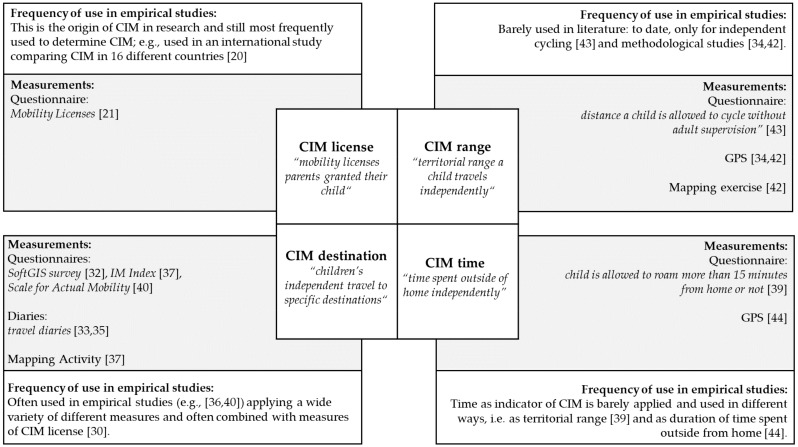
Definitions, measures, and frequency of application in empirical projects of four children’s independent mobility (CIM) indicators.

The term “children’s independent mobility” was introduced by Hillman et al. [21] in their seminal study “One false move…”in 1990, and is defined as “the freedom of children to travel around in their neighborhood or city without adult supervision” [22]. The definition of CIM is interpreted in different ways by different researchers: independent travel to a range of destinations (e.g., friend’s home, school, local shops, and playgrounds [23,24]), or explicitly, independent walking to/from school [19,25], independent travel as walking, cycling, and taking public transport without adult supervision [26,27], and sometimes also as independent play outside [27,28]. To better understand the multifactorial phenomenon of CIM, Figure 1 provides an overview of different CIM indicators and measurements, and their application in empirical studies.

### 2.1. Indicators of CIM

Based on Sharmin and Kamruzzaman [29], four indicators of CIM can be distinguished: CIM license, CIM destination, CIM time, and CIM range. CIM license describes the mobility licenses parents grant their child. As there might be a difference between where children are allowed to roam and where children actually roam, the second indicator CIM destination is used to determine the actual mobility of a child. This indicator is defined as children’s independent travel to specific destinations, such as local shops, school, and a friend’s house. Another indicator of children’s actual independent mobility is CIM range, reflecting the territorial range (usually expressed as the distance from a child’s home) a child travels independently. Additionally, time spent independently outside of home is used as another indicator of CIM. 

However, while these four indicators can theoretically be ascertained, in research practice, there is an overlap between them, mostly regarding CIM license and the other indicators. For example, parental questionnaires are used that evaluate parental allowance to travel to specific destinations independently [30]. This is due to the fact that parental licenses have a paramount role as an indicator for determining CIM. To date—given the heterogeneity of indicators, their overlapping, and the inconsistent state of research—the most relevant indicator of CIM in relation to effects on children’s health and development remains to be determined.

### 2.2. Measurements of CIM and Their Application in Empirical Studies

Typically, a questionnaire with six core questions for adults and children is used to measure CIM license (e.g., “Is your child allowed to cross main roads alone?” and “Is your child usually allowed to go out alone after dark?” [7,21]). Larouche et al. [31] confirmed this questionnaire as a reliable and valid tool to measure CIM. In order to determine the actual mobility of children, researchers from different countries introduced the use of mapping activities [19,32], travel diaries [33,34,35], go-along interviews [36], and other modified questionnaires [23,37,38,39,40]. With these methods, data on CIM destination and CIM range can be collected. With objective measures such as global positioning systems (GPS), distances from home (CIM range), and the relative time spent in the neighborhood (CIM time) were evaluated [33]. Additionally, single questions were used to determine CIM range (e.g., “How far from home are you allowed to roam on your own?” [39]).

In their methodological review, Bates and Stone [41] summarized the various methodological approaches used to determine CIM. The authors pointed out that studies on independent mobility used a variety of subjective (e.g., questionnaires, interviews, and travel diaries) and objective (e.g., accelerometer, GPS, and direct observation) measurements. Nevertheless, in this review, a distinction between the measurements of CIM according to the four indicators is not made. By reviewing the actual literature on CIM, empirical studies considering CIM license (e.g., References [20,21]) and CIM destination (e.g., References [32,37,40]) were mostly found, followed by CIM range (e.g., References [42,43]). The use of time to determine CIM is uncommon in empirical studies [39,44].

Bates and Stone [41] highlighted that only a few studies used similar methodologies, and that a standardized methodological approach does not exist. Most researchers used questionnaires (e.g., References [28,35,37,40,45]) and only a few used objective measurements (e.g., References [33,42,46]). Designing a comprehensive mixed-methods methodological approach would improve the comparison of studies [41] and help toward understanding the multidimensional phenomenon of independent mobility [46].

To overcome the lack of a standardized measurement protocol, Bhosale et al. [47] compared the traditional IM license (parental allowance to travel independently [21]) and the IM index (allowance to travel to certain destinations unsupervised [40]) with an interactive mapping activity including geographically defined data [48]. Although significant similarities were found between all three measures, the authors recommend a mixed-methods approach in future research that combines interactive mapping and the traditional parental and child surveys. Although, for children’s actively traveled distances, GPS-derived journeys might be the gold standard [42], GPS data are nevertheless challenging to handle and the devices could be expensive to purchase. Furthermore, ethical aspects such as the protection of privacy and data protection need to be considered as central barriers when planning a study applying GPS. Additionally, GPS data do not reflect the independence of a child from adult supervision. Thus, in any case, more information based on travel diaries or further questionnaires is required. Hence, a combination of objective (GPS) and subjective (questionnaires, travel diaries, and interviews) measurements should be considered in future research [41] to enable the evaluation of the four different indicators of CIM appropriately.

## 3. Children’s Independent Mobility as Health-Related Behavior

Although independent mobility does not necessarily reflect active behavior, it is mostly considered as independent active travel—like walking or cycling—without adult supervision [22]. In these cases, CIM is directly related to physical activity [49]. A systematic review on the association of CIM with physical activity, sedentary behavior, and weight status identified a consistent positive association between CIM and physical activity [49]. This was also confirmed in some more recent studies [5,26,27,50,51]. For both boys and girls, independently made trips are significantly associated with objectively assessed moderate-to-vigorous physical activity (MVPA) [44]. An increased percentage of daily trips that were made independently increased the time spent in MVPA daily. Additionally, CIM is positively related to accelerometer-measured weekday and weekend physical activity [51].

However, this positive association between CIM and physical activity can only be found if taking public transport unaccompanied is not taken into accounts. A study by Schoeppe et al. [27] considered walking, cycling, and taking public transport as independent travel, and found no association between CIM and physical activity. When excluding public transport, independent travel was positively associated with physical activity for boys. In adolescents, IM for walking and IM for cycling are related to non-school physical activity on weekdays, but not IM for taking public transport [26].

In their systematic review, Schoeppe et al. [49] were only able to include three studies considering CIM and health outcomes, and demanded further research on CIM and health-related outcomes, given that it remains unknown if more independent children are also less sedentary or overweight. A more recent study by Stone et al. [51] showed that children who are granted higher levels of CIM license spend less time being sedentary than those whose independent mobility is restricted. As physical activity and non-sedentary behavior are positively related to different health outcomes [52,53], CIM can be an important contributor to an active health behavior. Since an active lifestyle is often established in childhood and adolescence [54], necessity is given to promoting children’s active independent mobility in the local neighborhood early. Nevertheless, the long-term effects of CIM on physical activity later in life are yet to be examined.

In addition to physical activity and related health outcomes, CIM can contribute to children’s psycho-social and cognitive development [55,56], their social competencies [28], and their psychological wellbeing [57,58]. The independent interaction of a child with the environment while walking to school on their own provides better knowledge of environmental orientation and structure, as well as map reading [55,59]. Mackett et al. [56] pointed out that children walk slower when they are not under adult supervision, which may be associated with exploring the environment and socializing. In support of this notion, Prezza et al. [28] showed that children who gain higher levels of independent mobility play more often with peers than those who are less independent. Moreover, engaging in independent mobility in early childhood could reduce feelings of loneliness in adolescence [58]. The use of public open spaces, high levels of independent mobility, and consequently, a better relationship with the community in childhood lead additionally to an increased sense of community and reduced fear of crime in adolescence [58].

The benefit of CIM on children’s mental health is another important aspect to mention. Mobility restrictions [51], parental supervision while walking [56], or hyper-parenting [60] limit children’s physical activity. This physical inactivity could furthermore elevate the risk of depression [61]. Due to a lack of longitudinal studies, the long-term effects of independent mobility in childhood on preventing depression and promoting psychological wellbeing in adolescence or adulthood cannot be confirmed as of yet.

A case study in Hong Kong [57] considering short-term effects on children’s wellbeing during travel showed that children engaging in active transport rated their journeys happier than children who used motorized transportation. However, accompanied children rated their journey happier than children who were independently mobile. In this case, it is important to mention that more than 80% of the examined children were accompanied by an adult, and special focus was placed on primary school children. Thus, this study might only be representative for younger children that, in general, are less independently mobile than older ones. Additionally, primary school children might have greater fear of danger and strangers when traveling alone compared to their older counterparts, and thereby suppress feelings of wellbeing during travel. Further research is needed to explore the relationship of CIM with wellbeing in older age-groups that are, in fact, more independently mobile [20]. 

The lack of high-quality longitudinal studies focusing on CIM and health-related outcomes limits the evidence for causal relationships. Thus, the results should be interpreted carefully, and more studies employing a longitudinal design are required.

## 4. Prevalence and Trends in Children’s Independent Mobility

In a cooperative research project of the Policy Studies Institute London (PSI London, UK) CIM was compared in 16 different countries [20]. Using a translated version of the mobility licenses questionnaire for each participating country adopted from prior studies [7,21], the degree of traveling around independently in children between seven and 15 years of age was surveyed. The overall ranking showed that children in Finland enjoyed the greatest freedom to travel independently, followed by Germany, Norway, Sweden, Japan, and Denmark. Behind this group of high levels of independent mobility, England ranked at an average level. Less freedom of movement was found in France, Israel, Sri Lanka, Brazil, Ireland, and Australia. The lowest levels of independent mobility were registered in Portugal, Italy, and South Africa. Cultural attitudes and behavior, legal requirements, or road traffic rules and legislation of a country may affect these mobility licenses [20].

Generally speaking, girls have less freedom to travel around independently and become independently mobile later than boys [62]. However, findings concerning the influence of gender on CIM are inconsistent. In most countries—such as Germany, Finland, and Sweden—no significant difference was found between girls’ and boys’ mobility licenses [20]. In Italy, where parents granted their sons significantly more mobility licenses than their daughters, social danger perception of mothers was associated with their child being female [20,45], which might explain such appearing gender differences. Although CIM did not significantly differ between boys and girls in the majority of examined countries, promoting CIM should be considered—especially for girls. A recent study by De Meester et al. [50] identified CIM as an important mediator between parental perceived neighborhood attributes and physical activity for girls, and not for boys. As girls are generally less physically active than boys [2], encouraging girls to be independently mobile could help them comply with physical activity guidelines, such as the global recommendation on physical activity for health of the World Health Organization [1].

For all countries, an increase in CIM was reported by a child’s age [20]. Different stages of independence from adults that a child goes through are described in the literature [46,56]. When a child is young, it has round-the-clock adult supervision; afterward, a child may be allowed to go out with older siblings or friends, and, at some point, is granted full independence. For instance, in Portugal, about 13% of eight-year-old children are allowed to travel independently to local destinations, while, at the age of 15 years, more than 87% are allowed to go to local destinations on their own [24].

Especially in Finland, Germany, Norway, Sweden, Japan, and Denmark, children enjoy high levels of freedom to travel around independently [20]. Nonetheless, the percentage of children to which mobility licenses are granted decreased over the last 20 to 30 years [6,7,8], as presented in Table 1. A study by Kyttä et al. [6] identified a significant decline in CIM over two decades. In Finland, the number of children engaging in at least one independent weekend activity dropped from 89% in 1990 to 65% in 2010. In Australia, the proportion of children traveling independently to school declined from 61% to 32% between 1991 and 2012 [8]. A comparative study of children in England and Germany showed that, from 1990 to 2010, for both countries, the level of independent mobility decreased significantly [7]. In 1990, about 90% of the German study population was allowed to travel home from school alone; by 2010, the percentage was around 76% for German children. Children in England were granted fewer mobility licenses compared to Germany. For English children, a decline of 10% in the proportion of children traveling home from school independently was reported over 20 years, resulting in a proportion of 25% of children traveling independently in 2010 [7]. To identify intergenerational changes in CIM, Bhosale et al. [63] asked parents in New Zealand to recall their independent travel behavior at the age of 10–12 years, retrospectively, and compared the findings with the actual mobility licenses of the children of the participating parents. The findings demonstrated significant intergenerational differences in parental permissions to travel independently and the destinations independently traveled, with a decline in the younger generation. 

In literature, different reasons are mentioned for the remarkable decline in CIM over the years. A study by Fyhri et al. [12] highlighted that, in line with the decrease in independent walking and cycling, an increasing number of children were taken to school by car. More reasons for the decline include less walkable neighborhoods [10], parental safety concerns [13], busy family schedules [15], and longer distances from school and leisure activities [16]. 

In summary, these findings call for intervention programs promoting independent mobility and stopping the declines in independent mobility—especially in countries with low levels of CIM. Through the identification of determinants which inhibit or promote CIM and the development of an intervention program targeting these determinants, a further decline in children engaging in independent mobility should be prevented. 

## 5. Socio-Ecological Correlates of Children’s Independent Mobility

Health promotion programs are increasingly designed based on socio-ecological models [64,65]. The ecological perspective on health behavior change is based on four core principles: (1) multiple levels of factors influence heath behavior; (2) influences interact across levels; (3) multilevel interventions should be most effective in changing behavior; (4) ecological models are most powerful when they are behavior-specific [66] (p. 470). Thus, identifying various levels of contextual influence on CIM and their interaction is required. Numerous studies aimed to identify determinants that either promote or inhibit CIM (e.g., References [32,38,67,68]). However, these studies lack the appropriate study design to evaluate determinants of CIM and infer causal relationships [69]. Hence, according to Bauman et al. [70], until now, the factors associated with CIM needed to be considered as correlates due to the limited number of prospective studies.

**Figure 2:**
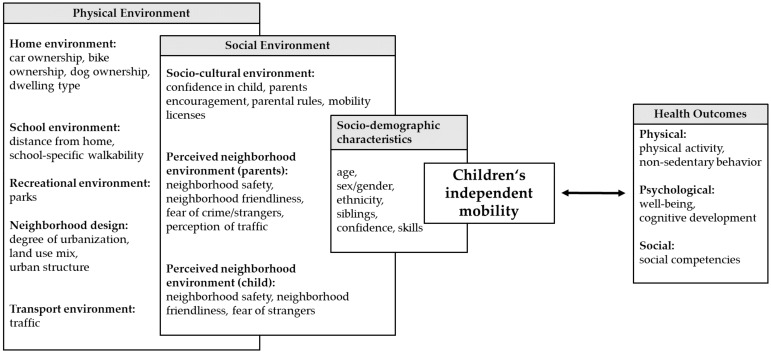
Summary of environmental correlates of CIM based on a socio-ecological perspective, according to Sallis et al. [72].

CIM is correlated with a number of socio-demographic, social, and physical environmental attributes as clearly demonstrated in recent systematic reviews [29,69,71]. An overview of multilevel correlates of CIM which are based on a social-ecological perspective [72] is provided in Figure 2.

Socio-demographic characteristics, such as age [19,24,35,45,56], gender [16,30,73], ethnicity [67,68], confidence and skills to travel independently [37,43], and older siblings [35,38], were consistently associated with the extent of CIM. Although many studies evaluated built environmental attributes, the evidence for associations with CIM is limited [69]. Multivariate regression models showed that, in addition to a child’s age and gender, social environmental attributes are often the only remaining significant factors associated with CIM [69]. Parents granting a child the freedom to travel around independently and parental perception of the neighborhood environment could be crucial in relation to CIM [69]. Thus, parents are important gatekeepers for a child’s independence and active mobility.

Nevertheless, a child’s view of the environment should not be disregarded. In a qualitative study with children and parents [15], children reported a wider range of safety concerns than parents, such as being attacked or bullied by older children, and other environmental factors like darkness and animals. To date, studies often only focus on parental perceptions of neighborhood environmental attributes, and the number of studies considering children’s views is limited [19,30]. More research is needed to evaluate whether parental perceptions of environmental attributes are of greater importance than the perception of children themselves. 

## 6. Future Directions and Practical Implications

In addition to children’s health, growth, and the development of personal responsibility, CIM can contribute to environmental sustainability (e.g., by a reduced car use and low monetary costs). Thus, CIM is an important issue for public health interventions.

Future research needs improved methodological approaches of CIM, as well as comparable measurements and prospective designs, to ensure standardization to enable a comparison of international studies, and to assess causal determinants rather than just associations between variables. To fully understand the indicators, further research is suggested that considers CIM as a multifactorial phenomenon (CIM license, CIM destination, CIM range, and CIM time). Mobility licenses are directly correlated with destinations to which a child travels independently and seemed to be associated with almost the same environmental attributes, such as parental perception of the environment [69]. Until now, little is known about CIM range and CIM time and how they are influenced by the environment [69]. A combination of CIM range and CIM time could be interesting to address in future studies. For example, a child could stay outside of home independently for a defined length of time in the defined vicinity of home, while another child could be allowed to travel further from home but for not as long. 

More studies evaluating simultaneous determinants at all ecological levels are also required. The explanation of contextual influences of CIM and the identification of promoters and inhibitors of CIM will support the development of intervention programs to deter or prevent the decline in CIM.

In general, the promotion of physical activity should not only consider organized physical activity (e.g., in sport clubs or at school). Although parents often support children taking part in organized leisure activities by chauffeuring, as a result, they simultaneously limit their children’s activity spaces and free movement in the local neighborhood [74]. As a significant domain of youth physical activity, the necessity is given to promote unorganized independent play and mobility in the local neighborhood [3]. Additional benefits of these contributors to physical activity are several health and social outcomes that children do not accumulate in organized leisure activities, such as environmental knowledge and independence from adults [51,55,56]. Furthermore, CIM could be a door opener to get access to physical activity facilities and locations [75], to socialize with peers [28], and to explore the environment [56]. 

Showing that the number of children who travel around the neighborhood independently declined significantly in various countries [6,7], the necessity for interventions is obviously given. However, only one intervention study which addressed the promotion of CIM can be found in literature [76]. The “we go to school alone” program by Prezza et al. [76] was evaluated in two districts of Rome, Italy. The program was prepared in various steps: (1) choosing meeting points and routes to school and identifying dangerous points; (2) finding collaborators (e.g., senior citizens, shop owners, and the local police) to supervise the meeting points and routes; (3) designing posters to be placed at the routes; (4) painting footprints on the sidewalks to mark the routes to school. In the operative phase, children who were allowed went to school on their own accompanied with schoolmates. The initiative affected children’s daily lives in only one district. Children were more independently mobile on the home–school journey, were taken by car less often, and increased their general level of IM in their neighborhood. No differences were found for the other district with the same intervention program. 

The authors explained the limited success by the different levels of support and collaboration offered by the local community’s social and political organizations. This highlights the importance to develop multilevel interventions that target the family and neighborhood, as well as multiple stakeholders. In comparison to CIM, many studies considered interventions on active transportation to and from school (e.g., the implementation of a walking school bus [77]). These studies reported a small, but promising, effectiveness in increasing active travel to school [77]. As CIM is a gatekeeper to active travel, similar effects might be found for interventions on CIM with additional benefits. The fact that only active, non-motorized independent mobility is associated with physical activity [26,27] and that children who walk on their own provide higher levels of physical activity than children who walk with adult accompaniment [56] leads to the conclusion that intervention programs should consider both independent mobility and active mobility. 

To promote independent mobility in the local neighborhood, child-friendly environments need to be created that enable children to travel around on their own. Child-friendly environments should alleviate parent and child concerns about traffic and strangers, and promote children’s possibilities to freely interact with their environment [40]. Especially for young children, activity spaces are needed that offer them the freedom to discover their physical opportunities and new environments [17]. Neighborhood friendliness, sense of community, and the accompaniment by friends or siblings instead of walking alone might also affect parental allowance to be independently mobile [78]. 

For the promotion of CIM, new mobile technologies should also be taken into accounts. Studies reported an increased parental feeling of safety if children took a mobile phone while roaming in the neighborhood independently [14,36]. Through a mobile phone, parents can easily get in contact to with their children or directly observe their children through GPS tracking. Although mobile phone use is positively associated with sedentary behavior [79], mobile phone ownership has no negative effect on CIM [7].

## 7. Conclusions

This narrative review established a comprehensive overview of CIM and identified current research gaps and future analysis to fully understand CIM. Associated with children’s personal development, as well as health and social integration in addition to environmental benefits, CIM is an important issue of public health. Practical implications for the development of intervention programs are provided based on a multilevel perspective to be most effective. As CIM is indeed linked with numerous environmental variables, the promotion of CIM needs to be considered on an individual, family, social, and policy level to prevent insufficient physical activity worldwide [80] via the mediating role of CIM [50] while promoting children’s health.

An important aspect regarding this overview of CIM is that the majority of findings presented in this paper are based on cross-sectional studies which evaluated associations between children’s independent mobility and health with social environment and physical environment, respectively. Thus, to date, only associations can be described without any causal relationships or mechanisms. More high-quality longitudinal studies are needed to fully understand the determinants and mechanisms of CIM.

## Figures and Tables

**Table 1:** Percentage of children being allowed to travel home from school alone in different countries over time [6,7,8,63]. CIM—children’s independent mobility.

Country	1971 (%)	1990 (%)	2010 (%)	Relative Differences in CIM (1990–2010) (%)
Germany ^1^ [7]		93	76	−18
England ^1^ [7]	86	35	25	−29
Finland ^2^ [6]		85	65	−24
Australia ^3^ [8]		68	31	−54
New Zealand ^4^ [63]		98	91	−7

^1^ only primary school children; ^2^ independent school travel; ^3^ years: 1991 and 2012; ^4^ intergenerational change (retrospectively, no year mentioned).

## References

[1] WHO (2010). Global Recommondation on Physical Activity for Health.

[2] Hallal P.C., Andersen L.B., Bull F.C., Guthold R., Haskell W., Ekelund U. (2012). Global physical activity levels: Surveillance progress, pitfalls, and prospects. Lancet.

[3] Schmidt S.C.E., Henn A., Albrecht C., Woll A. (2017). Physical Activity of German Children and Adolescents 2003-2012: The MoMo-Study. Int. J. Environ. Res. Public Health.

[4] Cooper A.R., Andersen L.B., Wedderkopp N., Page A.S., Froberg K. (2005). Physical activity levels of children who walk, cycle, or are driven to school. Am. J. Prev. Med..

[5] Marques E.A., Pizarro A.I., Mota J., Santos M.P. (2014). Independent mobility and its relationship with moderate-to-vigorous physical activity in middle-school Portuguese boys and girls. J. Phys. Act. Health.

[6] Kyttä M., Hirvonen J., Rudner J., Pirjola I., Laatikainen T. (2015). The last free-range children? Children’s independent mobility in Finland in the 1990s and 2010s. J. Transp. Geogr..

[7] Shaw B., Watson B., Frauendienst B., Redecker A., Jones T., Hillman M. (2013). Children’s Independent Mobility: A Comparative Study in England and Germany (1971–2010).

[8] Schoeppe S., Tranter P., Duncan M.J., Curtis C., Carver A., Malone K. (2015). Australian children’s independent mobility levels: Secondary analyses of cross-sectional data between 1991 and 2012. Child. Geogr..

[9] Barker J., Kraftl P., Horton J., Tucker F. (2009). The Road Less Travelled—New Directions in Children’s and Young People’s Mobility. Mobilities.

[10] Blinkert B. (2004). Quality of the City for Children: Chaos and Order. Child. Youth Environ..

[11] Funk W. (2008). Mobilität von Kinder und Jugendlichen. Langfristige Trends der Änderung Ihres Verkehrsverhaltens. Materialien aus dem Insitut für Empirische Soziologie an der Friedrich-Alexander-Universität Erlangen-Nürnberg.

[12] Fyhri A., Hjorthol R., Mackett R.L., Fotel T.N., Kyttä M. (2011). Children’s active travel and independent mobility in four countries: Development, social contributing trends and measures. Transp. Policy.

[13] Francis J., Martin K., Wood L., Foster S. (2017). ‘I’ll be driving you to school for the rest of your life’: A qualitative study of parents’ fear of stranger danger. J. Environ. Psychol..

[14] Bennetts S.K., Cooklin A.R., Crawford S., D’Esposito F., Hackworth N.J., Green J., Matthews J., Strazdins L., Zubrick S.R., Nicholson J.M. (2018). What Influences Parents’ Fear about Children’s Independent Mobility? Evidence from a State-Wide Survey of Australian Parents. Am. J. Health Promot..

[15] Crawford S.B., Bennetts S.K., Hackworth N.J., Green J., Graesser H., Cooldin A.R., Matthews J., Strazdins L., Zubrick S.R., D’Esposito F. (2017). Worries, ‘weirdos’, neighborhoods and knowing people: A qualitative study with children and parents regarding children’s independent mobility. Health Place.

[16] Fyhri A., Hjorthol R. (2009). Children’s independent mobility to school, friends and leisure activities. J. Transp. Geogr..

[17] Schmidt W. (1993). Kindheit und Sportzugang im Wandel: Konsequenzen für die Bewegungsförderung. Sportunterricht.

[18] Broberg A., Kyttä M., Fagerholm N. (2013). Child-friendly urban structures: Bullerby revisited. J. Environ. Psychol..

[19] Buliung R.N., Larsen K., Faulkner G., Ross T. (2017). Children’s independent mobility in the City of Toronto, Canada. Travel Behav. Soc..

[20] Shaw B., Bicket M., Elliott B., Fagan-Watson B., Mocca E., Hillman M. (2015). Children’s Independent Mobility: An International Comparison and Recommendation for Action.

[21] Hillman M., Adams J., Whitelegg J. (1990). One False Move: A Study of Children’s Independent Mobility.

[22] Tranter P., Whitelegg J. (1994). Children’s travel behaviours in Canberra: Car dependent lifestyles in a low density city. J. Transp. Geogr..

[23] Page A.S., Cooper A.R., Griew P., Davis L., Hillsdon M. (2009). Independent mobility in relation to weekday and weekend physical activity in children aged 10–11 years: The PEACH Project. Int. J. Behav. Nutr. Phys. Act..

[24] Cordovil R., Lopes F., Neto C. (2015). Children’s (in)dependent mobility in Portugal. J. Sci. Med. Sport.

[25] Carver A., Panter J.R., Jones A.P., van Sluijs E.M. (2014). Independent mobility on the journey to school: A joint cross-sectional and prospective exploration of social and physical environmental influences. J. Transp. Health.

[26] Garcia-Cervantes L., D’Haese S., Izquierdo-Gomez R., Padilla-Moledo C., Fernandez-Santos J.R., Cardon G., Veiga O.L. (2016). Physical activity coparticipation and independent mobility as correlates of objectively measured nonschool physical activity in different school grades: The UP&DOWN study. J. Phys. Act. Health.

[27] Schoeppe S., Duncan M.J., Badland H.M., Oliver M., Browne M. (2014). Associations between children’s independent mobility and physical activity. BMC Public Health.

[28] Prezza M., Pilloni S., Morabito C., Sersante C., Alparone F.R., Giuliani M.V. (2001). The influence of psychosocial and environmental factors on children’s independent mobility and relationship to peer frequentation. J. Community Appl. Soc..

[29] Sharmin S., Kamruzzaman M. (2017). Association between the built environment and children’s independent mobility: A meta-analytic review. J. Transp. Geogr..

[30] Villanueva K., Giles-Corti B., Bulsara M., Trapp G., Timperio A., McCormack G., Van Niel K. (2014). Does the walkability of neighbourhoods affect children’s independent mobility, independent of parental, socio-cultural and individual factors?. Child. Geogr..

[31] Larouche R., Eryuzlu S., Livock H., Leduc G., Faulkner G., Trudeau F., Tremblay M.S. (2017). Test-retest reliability and convergent validity of measures of children’s travel behaviours and independent mobility. J. Transp. Health.

[32] Broberg A., Salminen S., Kyttä M. (2013). Physical environmental characteristics promoting independent and active transport to children’s meaningful places. Appl. Geogr..

[33] Oliver M., Witten K., Kearns R.A., Mavoa S., Badland H.M., Carroll P., Drumheller C., Tavae N., Asiasiga L., Jelley S. (2011). Kids in the city study: Research design and methodology. BMC Public Health.

[34] Mavoa S., Oliver M., Witten K., Badland H.M. (2011). Linking GPS and travel diary data using sequence alignment in a study of children’s independent mobility. Int. J. Health Geogr..

[35] Johansson M. (2006). Environment and parental factors as determinants of mode for children’s leisure travel. J. Environ. Psychol..

[36] Chaudhury M., Hinckson E., Badland H., Oliver M. (2017). Children’s independence and affordances experienced in the context of public open spaces: A study of diverse inner-city and suburban neighbourhoods in Auckland, New Zealand. Child. Geogr..

[37] Villanueva K., Giles-Corti B., Bulsara M., Timperio A., McCormack G., Beesley B., Trapp G., Middleton N. (2012). Where Do Children Travel to and What Local Opportunities Are Available? The Relationship between Neighborhood Destinations and Children’s Independent Mobility. Environ. Behav..

[38] Christian H.E., Klinker C.D., Villanueva K., Knuiman M.W., Foster S.A., Zubrick S.R., Divitini M., Wood L., Giles-Corti B. (2015). The Effect of the Social and Physical Environment on Children’s Independent Mobility to Neighborhood Destinations. J. Phys. Act. Health.

[39] Veitch J., Carver A., Hume C., Crawford D., Timperio A., Ball K., Salmon J. (2014). Are independent mobility and territorial range associated with park visitation among youth?. Int. J. Behav. Nutr. Phys. Act..

[40] Kyttä M. (2004). The extent of children’s independent mobility and the number of actualized affordances as criteria for child-friendly environments. J. Environ. Psychol..

[41] Bates B., Stone M.R. (2015). Measures of outdoor play and independent mobility in children and youth: A methodological review. J. Sci. Med. Sport.

[42] Badland H.M., Oliver M., Duncan M.J., Schantz P. (2011). Measuring children’s independent mobility: Comparing objective and self-report approaches. Child. Geogr..

[43] Ghekiere A., Deforche B., Carver A., Mertens L., de Geus B., Clarys P., Cardon G., De Bourdeaudhuij I., Van Cauwenberg J. (2017). Insights into children’s independent mobility for transportation cycling-Which socio-ecological factors matter?. J. Sci. Med. Sport.

[44] Oliver M., Parker K., Witten K., Mavoa S., Badland H.M., Donovan P., Chaudhury M., Kearns R. (2016). Children’s Out-of-School Independently Mobile Trips, Active Travel, and Physical Activity: A Cross-Sectional Examination from the Kids in the City Study. J. Phys. Act. Health.

[45] Alparone F.R., Pacilli M.G. (2012). On children’s independent mobility: The interplay of demographic, environmental, and psychosocial factors. Child. Geogr..

[46] Mikkelsen M.R., Christensen P. (2009). Is Children’s Independent Mobility Really Independent? A Study of Children’s Mobility Combining Ethnography and GPS/Mobile Phone Technologies. Mobilities.

[47] Bhosale J., Duncan S., Stewart T., Chaix B., Kestens Y., Schofield G. (2017). Measuring children’s independent mobility: Comparing interactive mapping with destination access and licence to roam. Child. Geogr..

[48] Chaix B., Kestens Y., Perchoux C., Karusisi N., Merlo J., Labadi K. (2012). An interactive mapping tool to assess individual mobility patterns in neighborhood studies. Am. J. Prev. Med..

[49] Schoeppe S., Duncan M.J., Badland H., Oliver M., Curtis C. (2013). Associations of children’s independent mobility and active travel with physical activity, sedentary behaviour and weight status: A systematic review. J. Sci. Med. Sport.

[50] De Meester F., Van Dyck D., De Bourdeaudhuij I., Cardon G. (2014). Parental perceived neighborhood attributes: Associations with active transport and physical activity among 10–12 year old children and the mediating role of independent mobility. BMC Public Health.

[51] Stone M.R., Faulkner G.E.J., Mitra R., Buliung R.N. (2014). The freedom to explore: Examining the influence of independent mobility on weekday, weekend and after-school physical activity behaviour in children living in urban and inner-suburban neighbourhoods of varying socioeconomic status. Int. J. Behav. Nutr. Phys. Act..

[52] Larouche R., Saunders T.J., Faulkner G., Colley R., Tremblay M. (2014). Associations between active school transport and physical activity, body composition, and cardiovascular fitness: A systematic review of 68 studies. J. Phys. Act. Health.

[53] Carson V., Hunter S., Kuzik N., Gray C.E., Poitras V.J., Chaput J.P., Saunders T.J., Katzmarzyk P.T., Okely A.D., Gorber S.C. (2016). Systematic review of sedentary behaviour and health indicators in school-aged children and youth: An update. Appl. Physiol. Nutr. Metab..

[54] Telama R., Yang X., Viikari J., Valimaki I., Wanne O., Raitakari O. (2005). Physical activity from childhood to adulthood: A 21-year tracking study. Am. J. Prev. Med..

[55] Rissotto A., Tonucci F. (2002). Freedom of Movement and Environmental Knowledge in Elementary School Children. J. Environ. Psychol..

[56] Mackett R., Brown B., Gong Y., Kitazawa K., Paskins J. (2007). Children’s Independent Movement in the Local Environment. Built Environ..

[57] Leung K.Y.K., Loo B.P.Y. (2017). Association of children’s mobility and wellbeing: A case study in Hong Kong. Travel Behav. Soc..

[58] Prezza M., Pacilli M.G. (2007). Current fear of crime, sense of community and loneliness in Italian adolescents: The role of autonomous mobility and play during childhood. J. Community Psychol..

[59] Ahmadi E., Taniguchi G. (2007). Influential Factors on Children′s Spatial Knowledge and Mobility in Home–School Travel A Case Study in the City of Tehran. J. Asian Archit. Build..

[60] Janssen I. (2015). Hyper-parenting is negatively associated with physical activity among 7–12 year olds. Prev. Med..

[61] Mammen G., Faulkner G. (2013). Physical activity and the prevention of depression: A systematic review of prospective studies. Am. J. Prev. Med..

[62] Brown B., Mackett R., Gong Y., Kitazawa K., Paskins J. (2008). Gender differences in children’s pathways to independent mobility. Child. Geogr..

[63] Bhosale J., Duncan S., Schofield G. (2017). Intergenerational change in children’s independent mobility and active transport in New Zealand children and parents. J. Transp. Geogr..

[64] Kok G., Gottlieb N.H., Commers M., Smerecnik C. (2008). The ecological approach in health promotion programs: A decade later. Am. J. Health Promot..

[65] Golden S.D., Earp J.A. (2012). Social ecological approaches to individuals and their contexts: Twenty years of health education & behavior health promotion interventions. Health Educ. Behav..

[66] Sallis J.F., Owen N., Fisher E.B., Glanz K., Rimer B.K., Viswanath K. (2008). Ecological Models of Health Behavior. Health Behavior and Health Education: Theory, Research, and Practice.

[67] Janssen I., Ferrao T., King N. (2016). Individual, family, and neighborhood correlates of independent mobility among 7 to 11-year-olds. Prev. Med. Rep..

[68] Wolfe M.K., McDonald N.C. (2016). Association between Neighborhood Social Environment and Children’s Independent Mobility. J. Phys. Act. Health.

[69] Marzi I., Demetriou Y., Reimers A.K. (2018). Social and physical environmental correlates of independent mobility in children: A systematic review taking sex/gender differences into account. Int. J. Health Geogr..

[70] Bauman A.E., Sallis J.F., Dzewaltowski D.A., Owen N. (2002). Toward a better understanding of the influences on physical activity: The role of determinants, correlates, causal variables, mediators, moderators, and confounders. Am. J. Prev. Med..

[71] Qiu L., Zhu X. (2017). Impact of Housing and Community Environment on Childern’s Independent Mobility: A Systematic Literature Review. Int. J. Contemp. Archit. New ARCH.

[72] Sallis J.F., Cervero R.B., Ascher W., Henderson K.A., Kraft M.K., Kerr J. (2006). An ecological approach to creating active living communities. Annu. Rev. Public Health.

[73] Mitra R., Faulkner G.E.J., Buliung R.N., Stone M.R. (2014). Do parental perceptions of the neighbourhood environment influence children’s independent mobility? Evidence from Toronto, Canada. Urban Stud..

[74] Tillberg Mattsson K. (2002). Children’s (in)dependent mobility and parents’ chauffeuring in the town and the countryside. Tijdschr. Econ. Soc. Geogr..

[75] Reimers A.K., Wagner M., Alvanides S., Steinmayr A., Reiner M., Schmidt S., Woll A. (2014). Proximity to Sports Facilities and Sports Participation for Adolescents in Germany. PLoS ONE.

[76] Prezza M., Alparone F.R., Renzi D., Pietrobono A. (2010). Social participation and independent mobility in children: The effects of two implementations of “we go to school alone”. J. Prev. Interv. Community.

[77] Chillón P., Evenson K.R., Vaughn A., Ward D.S. (2011). A systematic review of interventions for promoting active transportation to school. Int. J. Behav. Nutr. Phys. Act..

[78] Christian H.E., Villanueva K., Klinker C.D., Knuiman M.W., Divitini M., Giles-Corti B. (2016). The effect of siblings and family dog ownership on children’s independent mobility to neighbourhood destinations. Aust. N. Z. J. Public Health.

[79] Barkley J.E., Lepp A., Salehi-Esfahani S. (2015). College Students’ Mobile Telephone Use Is Positively Associated With Sedentary Behavior. Am. J. Lifestyle Med..

[80] Guthold R., Stevens G.A., Riley L.M., Bull F.C. (2018). Worldwide trends in insufficient physical activity from 2001 to 2016: A pooled analysis of 358 population-based surveys with 1·9 million participants. Lancet Glob. Health.

